# Tumor Infiltration Levels of CD3, Foxp3 (+) Lymphocytes and CD68 Macrophages at Diagnosis Predict 5-Year Disease-Specific Survival in Patients with Oropharynx Squamous Cell Carcinoma

**DOI:** 10.3390/cancers14061508

**Published:** 2022-03-15

**Authors:** Borghild Ljokjel, Hilde Haave, Stein Lybak, Olav Karsten Vintermyr, Lars Helgeland, Hans Jørgen Aarstad

**Affiliations:** 1 Department of Otolaryngology/Head and Neck Surgery, Haukeland University Hospital, 5021 Bergen, Norway; borghild.ljokjel@helse-bergen.no (B.L.); hilde.haave@helse-bergen.no (H.H.); stein.lybak@helse-bergen.no (S.L.); 2 Department of Clinical Medicine, Faculty of Medicine, University of Bergen, 5021 Bergen, Norway; olav.vintermyr@helse-bergen.no (O.K.V.); lars.helgeland@helse-bergen.no (L.H.); 3 Department of Pathology, Haukeland University Hospital, 5021 Bergen, Norway

**Keywords:** human papilloma virus, oropharynx head neck cancer, T lymphocyte, T regulatory lymphocyte, macrophage, immunology

## Abstract

**Simple Summary:**

Head and neck cancer (HNC) is the sixth most common cancer worldwide, with a general prognosis of 50% disease-specific survival (DSS). The subgroup of oropharyngeal (OP) cancers are of interest because HPV infection is one of several causative agents and carries favorable prognosis. Influxes of inflammatory cells into tumors may vary with prognosis. T lymphocytes are important regarding specific immune defense. Within the immune system T regulatory cells (Foxp3 positive) co-governs this process. We have therefore primarily studied levels of Foxp3 (+) cells in malignant tumors from 170 patients related to prognosis of the patients. Higher levels of T lymphocyte Foxp3 (+) cells predicted better 5-year DSS. This case was unique relative to age, gender, TNM stage, and HPV infection; but more so among tumor HPV (+) than HPV (−) patients. The results encourage further study into the use of immune-based therapy in HNC patients.

**Abstract:**

Head and neck cancer (HNC) is the sixth most common cancer worldwide. Oropharyngeal (OP) cancers are of special interest because of possible underlying HPV infection which is tied to prognosis. Influxes of inflammatory cells into tumors may vary with prognoses. We wanted to study whether the number of tumor-infiltrating lymphocytes (TIL) and tumor-associated macrophages (TAM) in tumors correlated to HPV status and predicted 5-year disease-specific survival (DSS). Formalin-fixed paraffin-embedded (FFPE) biopsies cut sections from 170 patients treated for OP cancer were stained by immunohistochemistry and evaluated for the number of CD68 (+) TAMs, CD3 (+), and Foxp3 (+) (T regulatory) TILs. From FFPE slides HPV by PCR and p16 by immunohistochemistry were established. From FFPE Hematoxylin-Eosin slides, levels of tumor nuclear polymorphism, tumor invasion, desmoplasia, and inflammation were determined as previously published. Levels of TIL CD3 (+) and TIL Foxp3 (+) were increased among the HPV (+) compared to the HPV (−) patients. High levels of TIL Foxp3 (+) and CD68 (+) macrophages predicted better 5-year DSS. TIL Foxp3 (+) levels predicted independent of age, gender, TNM stage, and HPV infection as well as level of stromal desmoplasia, tumor invasion, and nuclear polymorphism, but more pronounced among tumor HPV (+) than HPV (−) patients.

## 1. Introduction

Head and neck cancer (HNC) is the sixth most common cancer group worldwide [1], and has a relatively grim prognosis. HNC kills worldwide about 50% of those who are diagnosed [2]. In the western world, one third of the patients diagnosed with tonsillar and base of the tongue (BOT) squamous cell carcinoma (SCC) succumb to the disease within five years [3]. It is of paramount importance to learn more about HN cancers. HNCs are, however, a heterogenic group of diseases with to some extent different causes, treatment, and prognosis with site of the disease as an important discriminator [4]. To simplify, we have therefore studied patients with cancers originating in oropharynx. An interesting and challenging aspect of SCC evolving with this origin is that many of these cancers are caused by HPV versus others that caused by tobacco and alcohol consumption [5].

Both epidemiological and experimental evidence suggest links between inflammation, specific immunity, and cancer, regarding both defense against [6], and establishment [7] of such diseases. This seems to be especially important when a virus causes cancer [8]. This should encourage the investigation of inflammation and immunity with respect to oropharynx squamous cell carcinoma (OPSCC) pathogenesis.

Levels of inflammatory cells within tumors predict prognosis in various malignant tumors [9].The number of both tumor-infiltrating lymphocytes (TIL) [10] and tumor-associated macrophages (TAMs) [11] has been tied to cancer prognosis. HNSCCs cancers may carry worse prognoses with high TAM numbers [12], whereas high TIL numbers have been associated with a better prognosis [13]. 

CD3 is a general marker for T lymphocytes [14]. The T cells are, e.g., responsible for cell-mediated specific immunity; their membrane-bound receptors can recognize antigens presented to them by antigen-presenting cells and start immune responses, which eliminate, e.g., virally infected cells or tumor cells. A class of T cells called T regulatory cells primarily mediate the suppression of the immune response. Tregs represent 5–10% of peripheral CD4 T cells [15]. “Repressive forkhead or winged-helix family transcription factor” (Foxp3) is one important marker of the T regulatory cells [16]. Foxp3 directly binds to the regulatory elements of IL-2 and IFN-γ genes and are supposed to induce active de-acetylation of histone H3, thereby inhibiting chromatin remodeling and controlling gene transcription. Foxp3 binds to GITR, CD25, CTLA-4 genes and increase histone acetylation and contributes to the increased expression of these proteins in Tregs [17]. Foxp3 also interacts with other transcription factors and is considered a master regulator of Tregs functions [16]. It has also been shown that levels of Foxp3 lymphocytes predict prognosis in some HNSCC [13]. In this study, we have evaluated Foxp3 TIL levels in OPSCC patients regarding prognosis. 

The mononuclear phagocyte (MNP) system includes monocytes, macrophages, and some dendritic cells [18]. MNP in tissue differentiate, e.g., to M1 or M2 macrophage with pro-inflammatory or anti-inflammatory properties, respectively [19]. M1 macrophages produce pro-inflammatory cytokines such as interleukin-1β (IL-1β), IL-6, IL-12, IL-23, and TNF-α; [19]. A large body of evidence supports the connection between IL-6 and cancer development [20]. In a review paper, the IL-6 serum level at diagnosis was, e.g., significantly correlated to survival in 82/101 series comprising 9917 out of 11,583 patients with 23 different cancer types [20]. The same is the case with HNSCC [21] patients. We [22,23] and others [5] have also shown an association between monocyte tumor reactivity and prognosis. We have therefore aimed at studying the level of macrophage infiltration as measured by CD68-positive cell levels in patients with OPSCC tumors. 

When measured from basic HE slides, the degree of stromal desmoplasia, the level of nuclear polymorphism, and invasion and lymphocyte infiltration relate to prognosis in OPSCC patients [24]. It should therefore be of interest to study any interaction to especially T lymphocyte Foxp3 level of infiltration and these parameters, and this has therefore formed a separate aim in the present investigation. 

The extent of disease, commonly described by the TNM stage of the disease [25], yields important prognostic information, and has therefore been included as important potentially interacting prognostic factors. Basic information about treatment, such as if treated with or without a surgery pillar [26], may also interact with the immunology of a disease. We have therefore included these dimensions to the study. 

We have aimed to study the five-year disease-specific survival (DSS) of newly diagnosed OPSCC patients dependent on the activation level of TILs measured by level of CD3 and Foxp3-positive TILs, as well as the level of tumor-associated macrophages (TAM) measured by CD68-positive cells. Furthermore, we have studied the TIL Foxp3 infiltration level five-year DSS adjusted by the degree of stromal desmoplasia, tumor lymphocyte infiltration, nuclear polymorphism, and degree of tumor invasion.

## 2. Materials and Methods

### 2.1. Patients

Since 1 January 1992 all patients diagnosed with head and neck cancer (HNC) at the Department of Otolaryngology/Head & Neck Surgery, Haukeland University Hospital (HUH), Bergen, Norway have been registered in a hospital-based HNC registry. HUH treats all cases of HNC in the Western Health Care Region of Norway. This region includes approximately one million inhabitants. 

All HN squamous cell carcinoma (HNSCC) patients were subjected to standardized diagnostic work-up, which consists of clinical examination, CT/MRI scans of the primary tumor site, neck, thorax, and liver, and ultra-sonographic examination of the neck including, if indicated, fine needle aspiration cytology. Diagnostic endoscopic examination under general anesthesia is performed if possible. From this population, patients with OPSCC diagnosed in the period from 1992 to 1 July 2008 were identified. 

### 2.2. Treatment

The treatment of the patient cohort from which the included OPSCC patients were drawn, have been published in detail [26]. In short, in the first period ranging from 1 January 1992 to 1 January 2000 the patients were primarily treated with radiation therapy (RT). From 1 January 2000 until 1 July 2008, the patients were treated primarily with surgery followed by postoperative RT. The five-year disease-specific survivals (DSS) were determined. 

### 2.3. Immunohistochemistry

Tumor tissue samples from oropharyngeal squamous cell carcinomas were fixed in buffered formalin and paraffin embedded. Sections were cut at 4 μm. The following antibodies were used in this study: Anti-CD68 (clone KP1, DAKO, dilution 1:3000), anti-CD3 (rabbit polyclonal, DAKO, dilution 1:100), and anti-Foxp3 (clone 259D/C7, BD Pharmingen, dilution 1:20). Antigen retrieval and staining were performed fully automated in a Ventana Benchmark Ultra according to the manufacturer’s instructions.

### 2.4. Scoring of Immunohistochemistry

**Figure 1:**
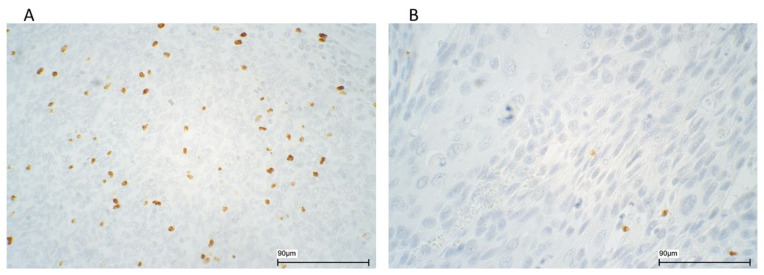
Immunohistochemical stainings of FOX P3 in HPV positive (**A**) and HPV negative (**B**) cases of oropharyngeal squamous cell carcinoma. Photomicrographs at ×400 magnification.

Part of the material was evaluated concomitantly in a two-headed microscope by the primary investigator (BL) and an experienced histopathologist (LH), the remaining by the primary investigator alone. Scoring was carried out by counting the number of positive cells per HPF at 630× magnification in a Zeiss Axio microscope, calculating the mean number of positive cells in five neighboring fields, randomly chosen. If the counts in two fields exceeded 200 cells, calculation of the mean number was based on those fields. The tumor stroma and tumor epithelium were evaluated separately and mean values calculated in each tissue compartment (Figure 1).

All sections were evaluated blindly, without knowledge of HPV status or histo-morphologic parameters as described below. An interim analysis was performed showing that all survival prognosis was tied to number of counted cells found in the tumor epithelium. Analyses from tumor neighboring fields were therefore discontinued, and the presented results are from tumor epithelial counting only.

### 2.5. Histological Evaluation

The histology grading has been scored as reported in detail previously [24]. The scoring system is given in Table S1. The morphologic features were given scores from 1 to 4 according to a histological grading system based on a publication from Kristensen et al. [27]. Fraction of mature cells was scored as percentage mature cancer cells and scored most (≥75%) to scarce (≤25%) and placed in 4 groups. Pattern of invasion was scored as 1. “pushing” with well delineated, infiltrating borders; 2. “infiltrating” with solid cords, bands, and/or strands; 3. “small groups or cords of infiltrating cells”; and 4. “marked and widespread cellular dissociation in small groups and/or single cell”. Tumor host inflammatory response was assessed as the degree of inflammatory cells around tumor cell islands and scored 1–4 depending on the presence of a marked, moderate, slight, or close to no inflammatory response. Tumor stromal desmoplasia was assessed as the degree of fibroblast response around tumor cell islands and scored 1–4 depending on the degree of the fibroblast response evaluated as close to none, slight, moderate, or marked, respectively.

### 2.6. DNA Isolation

All tumor samples were reviewed by an expert in pathology (OKV), and representative tissue samples were selected. DNA was extracted from formalin-fixed, paraffin-embedded (FFPE) specimens.

### 2.7. HPV DNA Detection

We have previously published this method in detail [28]. All other information from the patients was gathered and entered into the database without knowledge of the HPV status of the patients.

### 2.8. Statistics

The statistical program package PASW was employed (Ver. 26; SPSS Inc., Chicago, IL, USA) employing analyses as indicated. Results yielding *p* < 0.05 were considered statistically significant. All *p* values represent two-sided tests. Prognostic variables with disease-specific survival (DSS) were determined using a Kaplan–Meier estimator (log rank option) and/or Cox proportional hazards regression models. The five-year survivals are given as relative risk (RR) and confidence intervals (CI).

## 3. Results

### 3.1. Clinical Parameters

The present cohort (*n* = 170) represents a subgroup of the entire group of patients diagnosed in Western Norway with OPSCC (*n* = 280) in the period from 1992 until 1 July 2008. Table 1 shows the age, gender, tobacco history, TNM stage, and tumor site divided by tumor HPV status. The T and N stages five-year DSS are shown in Figures S1 and S2, respectively.

### 3.2. Morphological Evaluation of Tumor Host Responses

**Figure 2:**
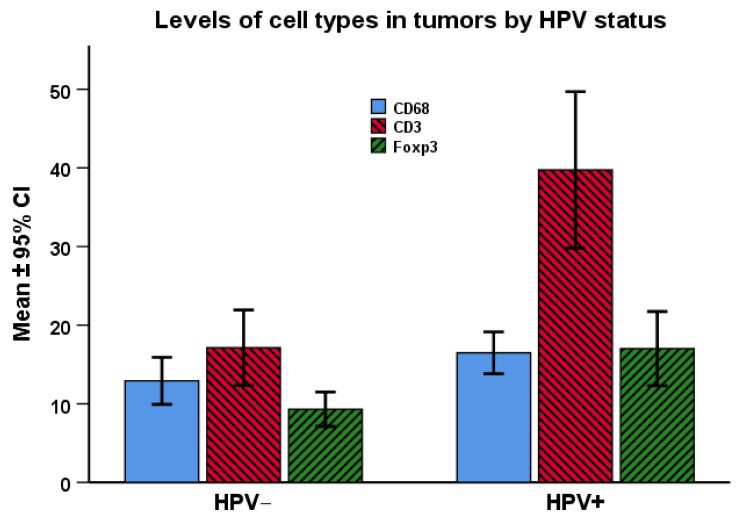
Cell type levels in tumors by HPV status, determined by PCR. Abbreviations: TAM: Tumor-associated macrophages (CD68 (+) cells). TIL: Tumor-infiltrating lymphocytes (CD3 (+)/Foxp3 (+) cells. These parameters determined by immunohistochemistry. Statistics by student *t*-test: CD68: *p* = 0.072; CD68-ln: *p* = 0.024. CD3: *p* < 0.001. Foxp3: *p* = 0.001. *Y*-axis: number of positive cells per HPF at 630x magnification in a Zeiss Axio microscope, calculating the mean number of positive cells in five neighboring fields, randomly chosen; mean ± confidence interval.

Magnitude of CD3-, CD68-, and Foxp3-positive cells divided by tumor HPV status are shown in Figure 2. Both levels of CD3- and Foxp3-positive TILs were increased among the HPV (+) compared to HPV (−) tumors; i.e., regarding CD3 43.1 ± 2.22 (mean ± SEM) versus 9.7 ± 1.32 (*p* < 0.001) and regarding Foxp3 17.7 ± 2.22 versus 9.7 ± 1.32 (*p* < 0.001). Regarding CD68-positive cells a trend was observed towards more cells among HPV (+) than HPV (−) tumors.

Table 2 shows correlation matrixes between the CD68, CD3, and Foxp3 values divided by HPV (−) and HPV (+) patients. The correlations between CD68 and CD3/Foxp3 values were significantly stronger among the HPV (−) than the HPV (+) patients (Table 2). 

Regarding the level of lymphocyte infiltration, the degree of desmoplasia, and nuclear polymorphism and invasion, significant positive correlations were shown to CD3 and CD68 levels. Regarding Foxp3 levels, the associations were more definitely shown among the HPV (−) patients than among the HPV (+) patients (Table 2).

### 3.3. Five-Year Disease-Specific Survival (DSS) Clinical Variables

**Figure 3:**
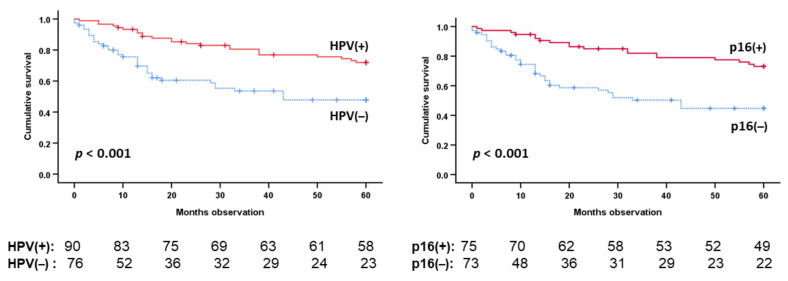
Five-year DSS (all patients) by tumor HPV (**left**) or p16 (**right**) status. HPV determined by PCR. p16 determined by immunohistochemistry. Two bottom lines each figure: Number patients at risk. Survival curves and statistics by Kaplan–Meier plot with Log rank *p* estimation.

Among the 170 included patients, 76 had tumors that were HPV (−) and 92 had tumors that were HPV (+). In addition, two patients had unknown HPV status. HPV infection status strongly predicted better five-year disease-specific survival (DSS) (*p* < 0.001) (Figure 3 left). p16 positivity also predicted better five-year DSS (*p* < 0.001) (Figure 3 right). Age of the patient at diagnosis predicted five-year DSS among HPV (+) (*p =* 0.005), but not among HPV (−) patients. The smoking history predicted five-year DSS among the HPV (−) patients. TNM stage predicted five-year DSS among the HPV (−) patients only. Five-year DSS was predicted by tumor site (tonsils/base of tongue versus elsewhere OP) among HPV (+) patients only (All Table 1).

### 3.4. Five-Year DSS by Kaplan–Meier Analyses Studying Dichotomized CD3, Foxp3 or CD68 Positivity

**Figure 4:**
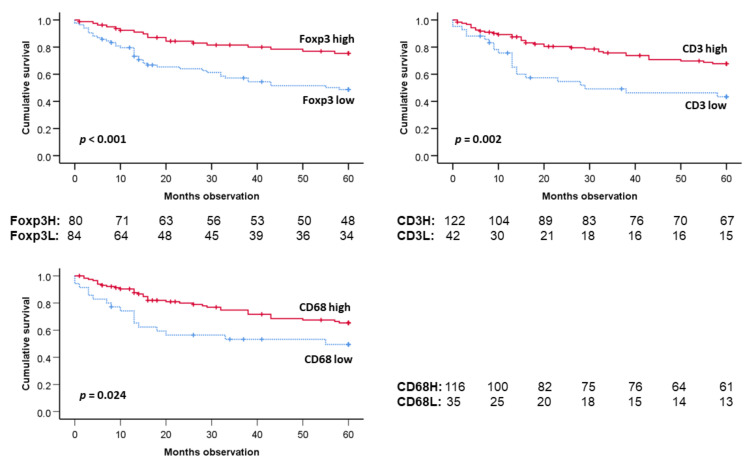
Five-year DSS (all patients) by TIL Foxp3 (+) (**top left**), CD3 (+) **top right**), or TAM CD68+ (**bottom**) status. CD3, Foxp3 and CD68 determined by immunohistochemistry. Two bottom lines on each figure: Number of patients at risk. Survival curves and statistics by Kaplan–Meier plot with Log rank *p* estimation.

We found that patients with a high tumor number of CD3-positive TILs had better five-year DSS than those with low such numbers (*p* < 0.05) (Figure 4). For Foxp3-positive TILs, it was shown that high numbers of TILs were tied to a better five-year DSS by Kaplan–Meier analyses (*p* < 0.001) (Figure 4). Concerning CD68 expression, i.e., TAM concentration, patients with such high numbers also had better five-year DSS than the patients with low TAMs (*p* = 0.025) (Figure 4).

**Figure 5:**
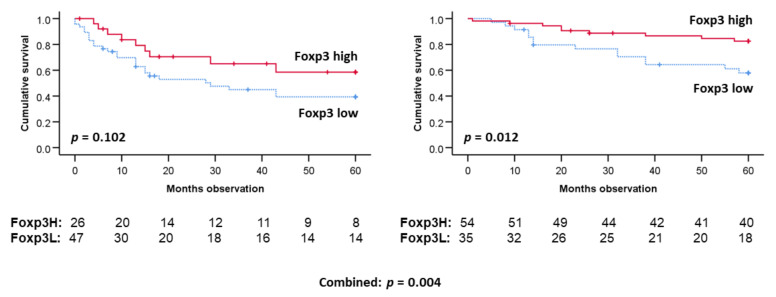
Five-year DSS TIL Foxp3 (+) levels by tumor HPV status. HPV determined by PCR. p16 determined by immunohistochemistry. Two bottom lines on each figure: Number of patients at risk. Survival curves and statistics by Kaplan–Meier plot with Log rank p estimation.

If the patients were stratified by whether they had HPV-infected tumors, five-year DSS prediction regarding Foxp3-positive TILs (*p* = 0.004) were still determined (Figure 5). Foxp3 TIL levels furthermore predicted survival among the HPV (+) patients only (*p* = 0.012). 

If the patients were stratified by CD68 density, the Foxp3 survival prediction was maintained (*p* = 0.007). If the patients were stratified by tumor median CD3 levels, the five-year DSS survival prediction by Foxp3 TIL levels were still valid (*p* = 0.007) (Results not shown).

### 3.5. Five-Year DSS by Cox Multivariate Regression Survival Analyses

If the results were studied by Cox regression survival analyses including gender, age, TNM stage, tobacco consumption history, and HPV status, the level of Foxp3 TIL remained a significant five-year DSS predictor (RR = 0.61; CI: 0.44–0.83) (Table 3). The levels of CD3- and CD68-positive cells did not remain predictors (Table 3). It was also studied the same survival prediction of HPV (+) or HPV (−) patients separately. In both cases, five-year DSS predictions by level of Foxp3 remained (Table 4).

### 3.6. Five-Year DSS Cox Multivariate Regression Analysis by Foxp3 Adjusted by Morphological-Derived Parameters with HPV Adjustment

Subsequently, we studied the same Cox regression as above cited, but with the addition of the various histological parameters. The Foxp3 survival prediction remained (Table 5). The same was the case when using a composite score based on summation of rate of desmoplasia and inflammation level among tumor HPV (+) but not HPV (−) patients (Results not shown).

### 3.7. Five-Year DSS Cox Stepwise Regression Analysis by Foxp3 TIL Levels, Age of the Patient, T Stage, and Whether HPV- and P16 Tumor Positivity

A Cox stepwise regression five-year DSS analysis was performed including age at diagnosis, T stage of tumor, Foxp3-positive TIL level, and whether HPV and/or p16 positive (Table 6). The results shows that the best explaining factor was tumor HPV, the second factor was the TIL Foxp3-positive level, and the third factor was age of the patient. Neither whether p16 positive nor T stage reached significance. This shows that the survival prediction from HPV status was better than from p16 analyses, and the p16 survival prediction is included within the HPV prediction (Table 6).

### 3.8. Five-Year DSS Cox Multivariate Regression Analysis Versus Treatment Period (RT vs. Surgery + RT)

Around the year 2000, the treatment strategy of OPSCC patients changed from mainly RT to surgery followed by RT. A Cox multivariate analysis was performed including HPV status, T stage, TIL Foxp3 positivity level, age of the patient, and whether treated in the first or last inclusion period of the study (Table 7). The results show that the treatment period did not matter regarding the five-year DSS which then shows that the Foxp3 TIL level prediction was the same regardless of including surgery as one major pillar of treatment or not.

## 4. Discussion

Levels of TIL CD3 (+)s and TIL Foxp3 (+)s were increased among the HPV (+) compared to the HPV (−) patients. High levels of TIL Foxp3, CD3 positive, and TAM (i.e., CD68+ cells) predicted better five-year DSS. This was the case with TIL Foxp3 levels unique relative to age, gender, TNM stage, HPV infection as well as rate of stromal desmoplasia, nuclear polymorphism, and tumor invasion.

Level of lymphocyte infiltration as measured by HE histology has been recognized as a positive marker for survival in OPSCC patients [29]. In both HPV (−) and HPV (+) HNSCC patients, high levels of TIL CD3 showed a positive prognostic value [13]. We have presently shown the same.

Foxp3 is a transcription factor belonging to the family of forkhead box (FOX) proteins. Foxp3 is associated with T lymphocyte development; in particular T regulatory lymphocytes (Tregs) [30]. It has also been shown that Foxp3 may be expressed in tumor cells [31]. In vitro studies have shown that Foxp3 can act as a tumor suppressor gene [32]. In breast cancer, Foxp3 have a tumor inhibitory role [31,33]. In prostate cancer, Foxp3 repress the transcription of c-MYC leading to inhibition of cell cycle progression and apoptosis [34]. In hepatocellular carcinoma, Foxp3 can suppress tumor progression via TGF-β/Smad2/3 signaling pathway [35]. In cervical cancer, silencing of Foxp3 downregulate p16INK4a, which inhibits cell proliferation [36]. Foxp3 may also function as promotor in tumor development. In lung adenocarcinoma, Foxp3 upregulates CCND1 (a gene of cell cycle G1/s checkpoint); thereby enhancing lung adenocarcinoma [37]. Thus, Foxp3 activation may both suppress and stimulate cancer cell growth. 

Tregs expressing Foxp3 are hypothesized to play an important part in immunological homeostasis and peripheral self-tolerance [15]. Tregs are recognized to harbor two major distinct populations, i.e., natural regulatory T cells (nTregs) differentiating in thymus and induced (i)Tregs formed associated with mucosa both in respiratory and GI mucosa [38]. The functions of nTregs and iTregs are to some extent different [39]. Tregs interact with many different immune cells including T and B lymphocytes, monocytes, macrophages, DC and mast cells [40]. Most of this interaction is supposed to be suppression, which is well described related to autoimmune disease [15]. 

Foxp3 TIL expression levels may also be associated with prognosis in cancer patients. High levels of Foxp3-positive lymphocytes have been linked to worse prognosis, e.g., colorectal cancer, melanomas, and lung carcinomas [41]. For other cancers, high levels of Foxp3-positive lymphocytes signal better prognoses, e.g., breast, prostate and gastric cancers. [41]. These dual findings are not easily in line with what is known about Tregs and autoimmune disease. Why it is so represents a major area where knowledge is lacking. 

In HNC, two different causes of cancer are determined with HPV or smoking/alcohol-related OPSCC [42] etiology. Possibly, Tregs interact differently with these two tumor entities. No conclusion regarding HPV (+) and HPV (−) OPSCC patients and prognostic relevance of Foxp3-positive Treg tumor TIL infiltration have so far been reached [13]. Our results support that high levels of positive TIL Foxp3s predict improved prognosis in both HPV (+) and HPV (−) patients, but more conclusively in HPV (+) than in HPV (−) OPSCC patients. 

The underlying causes as to why HPV-generated cancer exists remain unknown. One obvious suggestion is that the immune system in tonsillar and base of the tongue (BOT) tissue merit a privileged status for the epithelial cells [43]. This is illustrated by the fact that the HPV infection of these epithelial cells is more prone to generate carcinomas than at other OP sites [44]. 

The mechanisms underlying the carcinogenesis of the HPV virus are not fully known [45]. p16 induction is supposed to constitute a second step of HPV carcinogenesis following the primary step with deactivation of p53-dependent function [46]. Therefore, we have studied the level of p16-positive Foxp3-TILs as to DSS secondary to HPV-related DSS. Likewise, we have determined DSS predictions. We have also studied the importance of the TNM stage and shown that the prognosis of Foxp3 infiltration levels were not lost by the TNM stage adjustment. 

We have previously shown that a high degree of lymphocyte infiltration in OPSCC indicate better prognosis [24]. This is presently verified studying tumor CD3+ cells and Foxp3 (+) TILs. It is furthermore conceivable that more anaplastic cancer cells stimulate the immune system better through more neo-epitope generation. This is presently supported by a significant positive correlation between the level of nuclear polymorphism and Foxp3 TIL levels, but only in HPV (−) patients. We have, however, shown that the prognostic value of Foxp3 TIL levels were independent of nuclear polymorphism. The level of desmoplasia, i.e., fibroblast function [47], also predicts prognosis [24]. We have therefore compared the level of desmoplasia to the TIL Foxp3-positive levels. This was found regarding HPV (−) tumors. As with nuclear polymorphism, two unique dimensions of DSS-predicting measurements were found. 

One could hypothesize that the surgical removal of most of the tumor mass may tip the balance in the host towards the immune system than compared to relying only on RT-based tumor removal. We have therefore studied DSS in regard to whether the patients had been treated with surgical removal of the main tumor mass or not. We could not find any difference, and thus found no support in favor of such a hypothesis. 

In this study, the counting of the immunohistochemically stained slides were mainly carried out by one blinded person. More information could be gathered with morphometry, and this remains a future goal. The study reports from a cohort with limited number of patients. With more patients, survival results regarding HPV (+) patients in particular, could have been strengthened. The patients were from one hospital, and a multi-center study would be preferable. The patients were, however, drawn from most of the eligible patients from one geographical region and therefore are not strongly biased. Each patient has been closely followed up and therefore the results about the cause of death are accurate. The staining from all slides has been performed as a single procedure diminishing drift in the method over time. 

This study is one of several that encourages further studies about immune treatment of OPSCC. Immunotherapy is a therapy with potentially profound efficiency, but not all patients experience this, and this underlines the importance of choosing the correct patients [48]. Studying the TIL Foxp3 level in HNSCC patients eligible for immune therapy and correlating them to determine treatment results could be one future suggestion to better target such treatment. 

## 5. Conclusions

In conclusion, we have shown that high levels of CD3, CD68, and Foxp3 cells within the tumors of OPSCC patients better predict prognosis. The TIL Foxp3 prediction is best among HPV (+) tumors. The TIL Foxp3 survival prediction was to some extent independent of TNM stages, age, and gender as well as levels of tumor nuclear polymorphism, tumor invasion, desmoplasia, general lymphocyte infiltration, and TAM levels. The present results support the notion to carry out more studies about the Foxp3 system in OPSCC patients.

## Figures and Tables

**Table 1:** Clinical patient characteristics including patient age at diagnosis, site of tumor, smoking history, T and N stage as well as their mono-variate five-year disease-specific survival (DSS) all reported by HPV status (unknown for two patients).

Parameter	Tumor HPV (−)	Mono-Variate Cox Regression 5Y DSS Within HPV (−)	Tumor HPV (+)	Mono-Variate Cox Regression 5Y DSS Within HPV (+)
Age in years (Mean ± SD)	62.1 ± 11.0	n.s.	62.1 ± 11.0	*p* = 0.005
Gender (*n*; Male/Female)	63/13		65/27	n.s.
Tobacco history ^1^ (Mean ± SD)	3.5 ± 0.96	0.056	2.6 ± 1.36	n.s.
T stage ^2^ T1 T2 T3 T4	12 20 24 18	0.044	14 31 31 16	n.s.
N stage ^2^ N0 N1 N2 N3	29 14 25 5	<0.001	18 4 65 5	n.s.
M stage ^2^ M0 M1	68 3	0.004	92 0	n.a.
Total patients included	76		92	

^1^ Smoking scored as follows: I: Never smoked. II: Smoked less than 10 pack-years (twenty cigarettes per day for one year). III: Probably less than 10 pack-years. IV: Probably more than 10 pack-years. V: More than 10 pack-years. ^2^ TNM stage according to 7th TNM classification of malignant tumors by the Union for International Cancer Control.

**Table 2:** Pearson’s correlations between T and N stage, continuously measures levels of tumor epithelial CD3-, Foxp3-, and CD68-positive cells as well as HE histological malignancy grading; all by tumor HPV status.

Parameter	T Stage	N Stage	TAM CD68	TIL CD3	TIL Foxp3
Tumor HPV (−) patients					
TAM CD68	−0.22	0.07			
TIL CD3	−0.40 ***	0.14	0.62 ***		
TIL Foxp3	−0.21	0.12	0.49 ***	0.65 ***	
Nucl. Poly ^#^	−0.22	0.20	0.26	0.39 ***	0.38 **
Invasion ^#^	0.11	0.09	0.02	−0.30 *	**−0.22**
Inflammation ^#^	−0.19	−0.05	0.32*	0.49 ***	0.57 ***
Desmoplasia ^#^	0.31 *	−0.07	−0.20	−0.41 ***	−0.43 ***
Tumor HPV (+) patients					
TAM CD68	−0.04	0.05			
TIL CD3	−0.16	−0.01	0.28 *		
TIL Foxp3	−0.02	−0.03	**0.21**	0.42 ***	
Nucl. Poly ^#^	0.07	0.20	0.22	0.23 *	0.20
Invasion ^#^	0.18	−0.19	−0.16	−0.08	**0.04**
Inflammation ^#^	−0.29 **	0.29 **	0.21	0.22 *	**−0.16**
Desmoplasia ^#^	0.24 *	−0.12	−0.25 *	−0.29 **	**0.04**

*: *p* < 0.05. **: *p* < 0.01. ***: *p* < 0.001. Bold scores indicate statistical different correlation between HPV (−) and HPV (+) condition TNM stage according to 7th TNM classification of malignant tumors by the Union for international cancer control. TAM CD68: Tumor-associated macrophages levels according to rate of CD68 positivity (quartiles). TIL CD3/Foxp3: Tumor-infiltrating lymphocyte levels following staining with indicated epitope (quartiles). #: HE-based histological malignancy; 4-graded level.

**Table 3:** Cox multivariate regression five-year DSS including gender at diagnosis, age, TNM stage, tumor HPV status, tobacco consumption history as well as tumor-infiltrating levels of Foxp3, CD3 or CD68 measured from HE slides scored as quartiles. Showing only indicated covariate.

Covariate	Significance	RR	95% CI for RR	
			Lower	Upper
Including gender, TNM stage, HPV status and tobacco history + TIL Foxp3				
TIL Foxp3	0.002	0.61	0.44	0.83
Including gender, TNM stage, HPV status and tobacco history + TIL CD3				
TIL CD3	0.460	0.80	0.44	1.45
Including gender, TNM stage, HPV status and tobacco history + TAM CD68				
TAM CD68	0.058	0.54	0.28	1.02

RR: Relative Risk; CI: Confidence Interval. Abbreviations otherwise as in Table 1 and Table 2.

**Table 4:** Cox multivariate regression five-year DSS analysis including gender at diagnosis, age, TNM stage, tobacco consumption history and epithelial levels of Foxp3 (+) TILs measured as quartiles divided by tumor HPV status.

Parameter	Significance	RR	95% CI for RR	
			Lower	Upper
Tumor HPV (−) status				
Gender	0.238	0.38	0.08	1.89
Age of patient	0.196	1.03	0.98	1.09
T-stage	0.063	1.49	0.98	2.25
N-stage	0.003	1.91	1.30	2.92
M-stage	0.106	4.19	0.74	23.8
Tobacco history	0.107	1.74	0.89	3.40
TIL Foxp3	0.047	0.59	0.35	0.99
Tumor HPV (+) status				
Gender	0.980	0.99	0.29	3.38
Age of patient	0.001	1.08	1.03	1.12
T-stage	0.716	1.10	0.67	1.80
N-stage	0.105	1.65	0.90	3.00
Tobacco history	0.303	1.24	0.83	1.85
TIL Foxp3	0.044	0.66	0.44	0.99

Abbreviations as in Table 1, Table 2 and Table 3.

**Table 5:** Cox multivariate regression five-year DSS analyses including age of patient at diagnosis, TN stage, tumor-HPV status, and epithelial levels of Foxp3-positive cells and whether HE histology generated levels of desmoplasia, inflammation, nuclear ploidity, or invasion.

Covariate	Sign.	RR	95% CI for RR	
			Lower	Upper
Including patient age, TN-stage, HPV status + shown analysis from levels of TIL Foxp3 and desmoplasia				
TIL Foxp3	0.010	0.68	0.50	0.91
Desmoplasia	0.003	2.72	1.40	5.26
Including patient age, TN-stage, HPV status + shown analysis from levels of TIL Foxp3 and inflammation				
TIL Foxp3	0.003	0.63	0.46	0.86
Inflammation	0.500	1.27	0.63	2.55
Including patient age, TN-stage, HPV status + shown analysis from levels of TIL Foxp3 and polymorphism				
TIL Foxp3	0.000	0.56	0.42	0.76
Nucl. poly	0.019	2.30	1.15	4.59
Including patient age, TN-stage, HPV status + shown analysis from levels of TIL Foxp3 and level of invasion				
TIL Foxp3	0.001	0.61	0.45	0.82
Invasion	0.227	1.44	0.80	2.62

Abbreviations as in Table 1, Table 2 and Table 3.

**Table 6:** Cox stepwise regression five-year disease-specific survival analysis including age at diagnosis, T stage, epithelial levels of Foxp3-positive cells, HPV status and whether tumor p16 positive.

	Covariate	Significance	RR	95% CI for RR	
				Lower	Upper
Step 1	Tumor HPV	0.000	0.30	0.17	0.52
Step 2	Tumor HPV	0.001	0.37	0.21	0.65
	TIL Foxp3	0.002	0.64	0.48	0.85
Step 3	Tumor HPV	0.002	0.41	0.23	0.72
	Age of patients	0.028	1.03	1.00	1.06
	TIL Foxp3	0.001	0.612	0.46	0.82

Abbreviations as in Table 1, Table 2 and Table 3.

**Table 7:** Cox regression multivariate disease-specific five-year survival analysis including HPV tumor status, T stage, epithelial levels of Foxp3-positive cells and diagnosed 1992–1999 (treatment mainly RT only) versus 2000–2008 (treatment mainly surgery with postop RT).

Covariate	Sign.	RR	95% CI for RR	
			Lower	Upper
HPV	0.008	0.43	0.23	0.80
T-stage	0.171	1.21	0.92	1.59
TIL Foxp3	0.007	0.43	0.23	0.79
Age of patient	0.037	1.03	1.00	1.06
Treatment 1992–1999 vs. 2000–2008	0.824	0.93	0.52	1.70

Abbreviations as in Table 1, Table 2 and Table 3.

## Data Availability

The data from this study are not allowed to be share with anyone due to national legal obligations.

## Supplementary Materials

The following supporting information can be downloaded at: https://www.mdpi.com/article/10.3390/cancers14061508/s1, Table S1: histological grading of the tumors by Kristensen. Scoring system previously published [24,28]; Figure S1: five-year DSS by T stage divided into tumor HPV categories; Figure S2: five-year DSS by N stage divided into tumor HPV categories.
