# Supplementary material for: Tumor Infiltration Levels of CD3, Foxp3 (+) Lymphocytes and CD68 Macrophages at Diagnosis Predict 5-Year Disease-Specific Survival in Patients with Oropharynx Squamous Cell Carcinoma
Source: Cancers (Basel). 2022 Mar 15;14(6):1508. doi: 10.3390/cancers14061508 (PMC8946734; doi:10.3390/cancers14061508)
Supplement: Supplementary file 1 [file cancers-14-01508-s001.zip › cancers-1583135-supplementary.pdf]

Supplementary Information

Tumor Infiltration Levels of CD3, Foxp3 (+) Lymphocytes and CD68 Macrophages at Diagnosis Predict 5-year Disease-specific survival in Patients with Oropharynx Squamous Cell Carcinoma

Table S1. Histological grading of the tumors by Kristensen. Scoring system previously published [24, 28].

		HPV(-) *n* patients	HPV(+) *n* patients	Statistics HPV(-) vs. HPV(+) *p*-Value
		**Keratinization**		
1	>50%	9	2	
2	20-50%	18	7	
3	5-20%	23	17	
4	0-5%	15	57	<0.001
		**Cancer cell nuclear polymorphism**		
1	>75%	7	1	
2	50-75%	11	2	
3	25-50%	28	17	
4	0-25%	19	63	<0.001
		**Invasion**		
1	Pushing	1	20	
2	Infiltrating solid cords	22	39	
3	Infiltrating cells	24	17	
4	Widespread	18	7	<0.003
		**Inflammatory response**		
1	Marked	9	31	
2	Moderate	11	32	
3	Slight	39	16	
4	None	6	4	<0.001
		**Stromal desmoplasia**		
1	None	9	34	
2	Slight	17	23	
3	Moderate	22	21	
4	Marked	17	5	<0.001

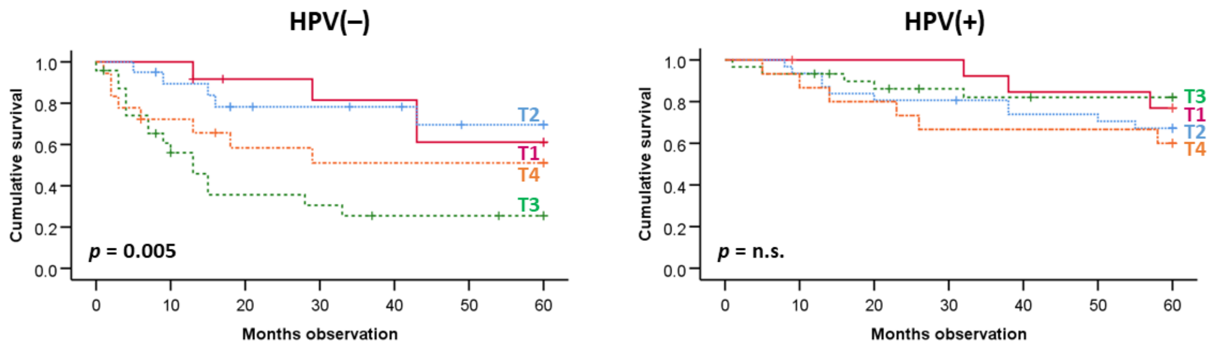

HPV(–):

	0	10	20	30	40	50	60
**T1:**	12	12	9	8	8	6	6
**T2:**	20	16	12	11	10	7	7
**T3:**	24	13	7	6	4	4	3
**T4:**	47	11	8	7	6	6	6

HPV(+):

	0	10	20	30	40	50	60
**T1:**	14	13	13	13	11	11	10
**T2:**	31	29	26	25	22	22	20
**T3:**	30	28	25	21	20	19	19
**T4:**	15	14	12	10	10	10	9

Figure S1. Five year DSS by T stage divided into tumor HPV categories. PCR determined HPV status and T stage from the international cancer union classification. Survival curves and statistics by Kaplan-Meier plot and Log-rank tests.

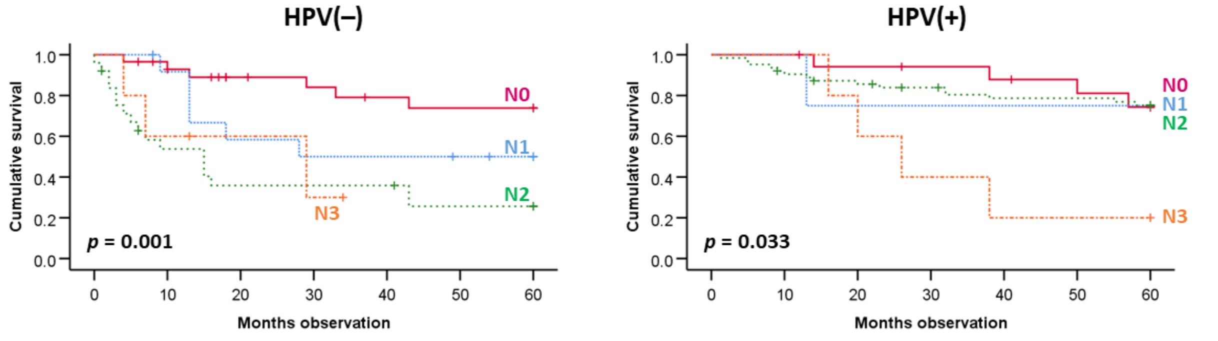

HPV(–):

	0	10	20	30	40	50	60
**N0:**	29	26	19	17	15	14	13
**N1:**	14	11	12	7	6	5	4
**N2:**	25	12	8	8	8	5	5
**N3:**	5	3	2	1	0	0	0

HPV(+):

	0	10	20	30	40	50	60
**N0:**	18	18	16	15	14	13	11
**N1:**	4	4	3	3	3	3	3
**N2:**	63	57	53	49	45	45	43
**N3:**	5	5	4	2	1	1	1

Figure S2. Five year DSS by N stage divided into tumor HPV categories. PCR determined HPV status and T stage from the international cancer union classification. Survival curves and statistics by Kaplan-Meier plot and Log-rank tests.
